# PROTOCOL: The association between marital transitions and physical and mental health in late life: A systematic review

**DOI:** 10.1002/cl2.1252

**Published:** 2022-06-08

**Authors:** Jing Li, Xu Hui, Zhenxing Lu, Xiaocao Ren, Wenlong Yan, Peijing Yan, Liang Yao, Kehu Yang

**Affiliations:** ^1^ School of Public Health, Evidence‐based Social Sciences Research Center, Health Technology Assessment Center Lanzhou University Lanzhou China; ^2^ Institute of Medical Research Northwestern Polytechnical University Xi'an China; ^3^ Clinical Medical College Lanzhou University Lanzhou China; ^4^ Department of Health Research Methodology, Evidence and Impact McMaster University Hamilton Canada

## Abstract

This is the protocol for a Campbell systematic review. The objectives are as follows: What is the association between marital transitions and physical health among people older than 60? What is the association between marital transitions and mental health among people older than 60? What is the role of gender, age, and education on the association between marital transitions and health among people older than 60? What is the influence of geographical region, housing, neighborhood, and social support on the association between marital transitions and health status among people older than 60?

## 1 BACKGROUND

### 1.1 The problem, condition, or issue

The world population is rapidly aging. Today, for the first time in history, most people can expect to live into their sixties and beyond. By 2050, the world's population aged 60 years and older is expected to total 2 billion, up from 900 million in 2015 (WHO, 2018). The older people tended to have more complex health needs with a higher risk of comorbidity (≥2 concurrent diseases) (Pickard, 2018), they face special physical and mental health challenges which need to be recognized (WHO, 2017).

Common conditions in older age include hearing loss, cataracts, and refractive errors, back and neck pain and osteoarthritis, chronic obstructive pulmonary disease, and diabetes (Jaul & Barron, 2017; WHO, 2018). A study data from China Health and Retirement Longitudinal Study involving 6754 subjects, the results showed that 42% people had at least one chronic disease, 17% had two or more diseases at the same time; the top three diseases of prevalence were hypertension (15.4%), arthritis or rheumatism (11.0%), and stomach or digestive diseases (9.3%) (Fan et al., 2021). The data from the National Health and Nutrition Examination Survey from 2005 through 2012 showed that the prevalence of multimorbidity in adults aged 65 or older was 67%, especially in hypertension and arthritis (Jindai et al., 2016). A study based on the Longitudinal Survey on Senior's Health and Health Services Use linked to health‐Adm data, the result showed that multimorbidity was very high in old people in Quebec, with an estimated prevalence of up to 95.9% (Gontijo Guerra et al., 2019). A systematic review included a total of 52 articles (45 studies) that reported data among >60 million older people in 30 higher‐income countries, the overall prevalence of multimorbidity was 66.1% (interquartile range [IQR]: 54.4–76.6) (Ofori‐Asenso et al., 2018). Mental health has an impact on physical health and vice versa. For example, the older people with physical health conditions such as heart disease have higher rates of depression than those who are healthy (National Institute of Mental Health, 2018). Additionally, untreated depression in an older people with heart disease can negatively affect its outcome (WHO, 2017). Overlap of depression and anxiety is very common with up to almost half of the older people reporting significant depressive and anxiety symptoms (WHO, 2017). Also, depression is the most common psychiatric disorder in late life (Parkar, 2015).

Beyond biological changes, ageing is also associated with other factors such as marital transitions (In our study, marital transitions were defined as becoming divorced, widowhood, and remarriage [Carr, 2015; Eng et al., 2005].), retirement, relocation to more appropriate housing, and receive social support (Smyer, 1984). As the average life span lengthens, and the narrowing of the gap between male and female about life expectancy in most low‐mortality countries in the last few decades (Thorslund et al., 2013), the marital status composition of the older people has shifted in recent years (Recksiedler & Stawski, 2019), and the evidence about the association between marital transitions and physical and mental health in late life were inconsistent (Rendall et al., 2011).

### 1.2 The intervention

“Marital status” was defined as “the status of being married, single (remain unmarried), widowed, divorced or separated” (Eurostat Statistics Explained, 2019; Webster, 2021). Marital transition was defined as a change in marital status during a given time period that occurs due to entry into marriage, exit out of marriage as a result of divorce or spousal death, and remarriage following divorce or spousal death. Transitions into marriage could have included never married to married, divorced/separated, or widowed to remarried. Transitions out of marriage could have included cohabiting to not living with a romantic partner, married to divorced/separated, and married to widowed (Dinour et al., 2012). Numerous studies explored the relationship between marital status and health in late life, including original studies (Saito et al., 2017; Va et al., 2011) and systematic reviews (Kojima et al., 2020; Manzoli et al., 2007), but the limited evidence to explore the impact of marital transitions on health, therefore, our study focus on marital transitions.

Divorce refers to the legal termination of marriage, whereas separation refers to the point in a relationship when spouses stop living together due to difficulties in their marriage (Carr, 2015; Webster). In our study, the divorce and separation are treated as divorce events. From the 1990s to 2016, the prevalence trend of divorce among USA adults 50 years or older has risen, the divorce rate about 55–64 years old was 43% for both sexes, and those 65–74 years was 39%, which was still higher than for the general adult population (Brown & Wright, 2017; US Census Bureau, 2016). By 2030 the number of the older people experiencing divorce is estimated to grow by one‐third merely due to the larger size of the older adult population (Brown & Lin, 2012). Consistent with the rising in the divorce rate, today's older adults were more accepting of divorce than their predecessors were two decades ago. Attitudinal change was modest between 1994 and 2002 but accelerated after 2002 (Brown & Wright, 2019).

Widowhood, includes persons who have lost their legally‐married spouse through death and have not remarried (Statistics Canada, 2016). Widows (females whose spouse has died) and widowers (males whose spouse has died) may grieve and mourn their loss for years (CliffsNotes, 2020). Widowhood fell slightly among men from 7.5% in 1990% to 5.7% in 2015. The decline was sharper for women, whose levels of widowhood plummeted from 31.6% to 18.9% (Brown & Wright, 2017).

Remarriage is a marriage that takes place after a previous marital union has ended, as through divorce or widowhood (The Free Dictionary, 2021). Older adults, particularly women, are less likely than their younger counterparts to remarry following divorce and, to a lesser degree, the death of a spouse, which is due to fears of social disapproval, financial concerns, and the opposition of other family members. Among men and women 60 to 69 years old in USA, 23% had married twice and less than 10% had married three times or more (US Census Bureau, 2016). Increases in the number of older adults, increases in those who are single or divorced, longer life expectancies, and changes in values of the baby boomers entering retirement age portend that remarriage will occur more frequently in the future (Marriage and Family Encyclopedia, 2021; Navigating the Aging Process, 2009).

### 1.3 How the intervention might work

Health behavioral factors may underlie health and mortality differentials by marital status. Generally, married persons have healthier lifestyles than unmarried persons. Cross sectional evidence showed that married persons were more likely to quit smoking (Tillgren et al., 1996; Waldron & Lye, 1989) and less likely to drink excessively than unmarried persons (Broman, 1993; Luoto et al., 1998). Marriage has also been associated with higher body weight (Cairney & Wade, 1998; Sobal et al., 1992) and levels of physical activity (Osler, 1995; Schone & Weinick, 1998) but not consistently (Braddon et al., 1986; Shah et al., 1989). Unmarried persons, particularly solitary men, have poorer quality of diet including lower consumption of fruits and vegetables (Billson et al., 1999; Shahar et al., 2001).

There are several underlying mechanisms by which change in marital status may affect health behaviors. It has been hypothesized that the marital relationship provides social control (Social control is the study of the mechanisms, in the form of patterns of pressure, through which society maintains social order and cohesion) over health behaviors (Carmichael, 2018; Umberson, 1987). Social support from a spouse may also be a key mediating factor in the establishment and maintenance of a healthy lifestyle (Joung et al., 1995; Umberson, 1992). Psychological factors (e.g., depression), stress levels, and economies of scale represent additional pathways.

At age 60, divorced men have much greater odds of consuming 3 or more drinks per day than married men (Reczek et al., 2016). It is probable that changes in both social support and stress levels underlie the observed relations with marital termination. Smoking and heavy drinking are each related to high levels of stress (Lawless et al., 2015; Thørrisen et al., 2019) and low social support (Rostami et al., 2013). In fact, spousal support may buffer against stress (Choi et al., 2015) and thereby lead to reductions in smoking and drinking (Waldron & Lye, 1989). High levels of partner support have been prospectively associated with smoking cessation in treatment programs (Soulakova et al., 2018). Smokers who relied on social support from friends and family had higher odds of intending to quit than those who did not (odds ratio [OR] = 1.39, 95% confidence interval [CI] = 1.22:1.58).

Previous studies have also detected associations between marital break‐up and weight loss, as well as between marriage and weight gain (Sobal et al., 1992; Teachman, 2016; Wilson, 2012). Men who became divorced or widowed had respective BMI decreases of −0.31 (*p* < 0.0001) and −0.35 kg/m^2^ (*p* < 0.0001), relative to change in men who stayed married (Eng et al., 2005). Time demands of a new spousal role may preclude routine exercise (Sobal et al., 1992). Married life may also bring regularity to meal patterns (Sobal et al., 1992), and increased food intake via social facilitation (de Castro, 1994).

An European Prospective Investigation into Cancer (EPIC)Norfolk cohort conducted in UK explored the marital transitions and associated changes in fruit and vegetable intake (Vinther et al., 2016). The results showed that in 3.6 years of follow‐up and relative to men who stayed married, widowed men showed significant declines (mean difference [MD], 95% CI) in all four indicators of healthy eating including fruit quantity (47.7, 80.6 to 14.9 g/day), fruit variety (0.6, 1.1 to 0.2 no/month), vegetable quantity (27.7, 50.5 to 4.9 g/day), and vegetable variety (1.6, 2.2 to 0.9 no/month). Men who were separated or divorced or who remained single also showed significant declines in three of the indicators. Among women, only those who became separated/divorced or stayed single showed declines in one indicator, vegetable variety (Vinther et al., 2016).

### 1.4 Why it is important to do this review

Marital dissolution in later life, whether through divorce or widowhood, has been described as among the most stressful of all life transitions (Carr, 2015).

Some studies have shown a positive impact of marriage on health in the older people (Becker et al., 2019; Myroniuk, 2017; Wood et al., 2019), known as the marriage protection effect (MPE). For example, several studies reported that divorced individuals (males and females) were at higher risk of having dementia and stroke compared to married ones (Andersen & Olsen, 2018; Liu et al., 2020).

However, other studies showed that marriage can have a negative impact (Kutob et al., 2017) or no influence (Rendall et al., 2011) on health status, especially in different genders (Spahni et al., 2016), educational backgrounds (Murakami et al., 2017), and countries (Fu & Noguchi, 2016). Domestic violence is a major cause of unhappy marriages, involves kicking, biting, beating, choking, threatening to use or using a gun or knife, or sexual assault, a divorce can help you escape and live without fear (Grande et al., 2003; Vu et al., 2014). Some studies show that the benefits conferred by education are not necessarily ubiquitous, and its impact on the adaptation to different marital status and diseases are different (Murakami et al., 2017; Recksiedler & Stawski, 2019). Moreover, effects may vary by country of residence due to the different social and cultural habits of each country (Fu & Noguchi, 2016; Zheng et al., 2014).

A systematic review in 2009 explored the correlation between health status and relationship dynamics of late‐life couples (Walker & Luszcz, 2009). However, it suggested that research was needed to focus on several gaps in the literature, including the impact of housing, neighborhood, and social support on the link between marital transitions and health status, which was poorly addressed in the literature.

To the best of our knowledge, there is no systematic review investigating the association between marital transitions and physical/mental health in the older people. Therefore, to provide more reliable evidence to promote their quality of life, we will conduct a review of the association and test the role of gender, education, and other factors on this association.

## 2 OBJECTIVES

1. What is the association between marital transitions and physical health among people older than 60?
2. What is the association between marital transitions and mental health among people older than 60?
3. What is the role of gender, age and education on the association between marital transitions and health among people older than 60?
4. What is the influence of geographical region, housing, neighborhood and social support on the association between marital transitions and health status among people older than 60?

## 3 METHODS

### 3.1 Criteria for considering studies for this review

#### 3.1.1 Types of studies

Our study looking at observational studies as experimental interventions are not possible on this topic, because marital transitions cannot be the object of experimental interventions.

Inclusion criteria:

1 Case‐control studies, cohort studies and cross‐sectional studies that investigated marital transitions for physical and mental health.
2. Studies that report relative risk (RR) or OR with the corresponding 95% CIs or that offer data to perform the calculation.

Exclusion criteria:

We will exclude (1) literature reviews, editorial pieces, letters, case reports, and notes; (2) qualitative studies.

#### 3.1.2 Types of participants

We will focus on marital transitions which occur after the age of 60. Studies that also include younger adults (<60 years of age) will only be included if: they report the results separately for ≥60‐year‐olds, or they specifically define the average age of the study participants exceeds the 60.

#### 3.1.3 Types of interventions

Marital transitions over the age of 60, for example, becoming married, widowed, divorced/separated, or remarried.

#### 3.1.4 Types of outcome measures

Studies will be included if they have measured the effect of marital transitions on physical/mental health in late life. We will extract 2 × 2 tables, RR or OR‐related estimates—depending on the data availability in each study. When data are available as a point estimate (95% CI) derived from two or more multiple regression models, we will extract the estimates from the most complete model (full model).

##### Primary outcomes

We are interested in any measures of health that reported in the original study.

1. Physical health: such as stroke, hypertension, dementia, mortality, visual impairment, obesity, and etc.
2. Mental health: such as depression, anxiety, life satisfaction, loneliness, and etc.

##### Secondary outcomes

None.

#### 3.1.5 Duration of follow‐up

We will include all follow up durations at screening stage.

#### 3.1.6 Types of settings

None.

### 3.2 Search methods for identification of studies

We will search for all published studies in the most common medical databases. We will include Google Scholar searcher to retrieve additional studies and relevant references from related systematic reviews.

#### 3.2.1 Electronic searches

The following databases will be searched from inception to present:

1. MEDLINE (PubMed interface);
2. Embase (Embase.com interface);
3. Social Sciences Citation Index (Web of Science);
4. Applied Social Sciences Index and Abstracts (ASSIA);
5. International Bibliography of the Social Sciences (IBSS);
6. PsycInfo and PsycArticles (psycnet.apa.org);
7. ProQuest Sociology Database;
8. Scopus;
9. Academic Search.

The strategy will be tailored to each specific database and will comprise both index terms (when relevant; e.g., MeSH terms, Emtree terms) and free text words (in title or abstract), with attention to possible synonyms, spelling variants, and correct use of truncation and proximity operators. Search filters will not be used, as they may prevent the retrieval of relevant papers.

De‐duplication of the references will be done using the EndNote reference management software (EndNote, 2013). All searches and search dates will be documented. The search strategy for MEDLINE (PubMed interface) is provided in the Supporting Information: Appendix 1.

#### 3.2.2 Searching other resources

Grey literature sources and handsearching

We will consult the following sources of grey literature, and hand search the Campbell Systematic Reviews journal and the reference lists of any relevant systematic review. The hand search will focus on editions published from inception to 2022 to secure recently unpublished articles which have not yet been indexed in the bibliographic databases. Searching the following websites devoted to the specific topics of marital transitions and ageing, to identify relevant unpublished studies and reports:

1) Gray Literature Report (www.greylit.org);
2) OpenGrey (www.opengrey.eu);
3) ClinicalTrials.gov (clinicaltrials.gov);
4) International Clinical Trials Registry Platform of the World Health; Organization (ICTRP, apps.who.int/trialsearch/Default. aspx);
5) Google Scholar (scholar.google.com).
6) Age UK (www.ageuk.org.uk/our-impact/policy-research/publications/);
7) Centre for Ageing Better (www.ageing-better.org.uk/publications);
8) International Longevity Centre UK (ILCUK, ilcuk.org.uk/reports/);
9) WHO Ageing and life‐course Program (www.who.int/ageing/data-research/en/); The health and relationship dynamics of late‐life couples: a systematic review of the literature.
10) National Ageing Research Institute (NARI) in Victoria, Australia (www.nari.net.au/publications/overview-about-publications).
11) Some related ageing cohorts: The English Longitudinal Study of Ageing (ELSA); Healthy Ageing and Biomarkers Cohort Study (HABCS); The longitudinal urban cohort ageing study (LUCAS); The Korean Frailty and Aging Cohort Study (KFACS) and et al.

##### Other reviews

We will also search the reference lists of previous systematic reviews for additional studies (Dinour et al., 2012; Manzoli et al., 2007; Sommerlad et al., 2018; Walker & Luszcz, 2009).

##### Contact to experts

We will contact international experts to identify unpublished and ongoing studies.

### 3.3 Data collection and analysis

#### 3.3.1 Description of methods used in primary research

None.

#### 3.3.2 Selection of studies

Two review authors will select studies independently in Rayyan (https://www.rayyan.ai/). In a first phase, titles and abstracts of the references identified by the search will be screened. Full texts of potentially relevant papers will be retrieved, and references that meet the selection criteria will be included for further analysis. In addition, relevant conference abstracts will be included to identified full text. Studies that meet the selection criteria and had the outcomes of interest measured, but do not report these outcome data, will be excluded from the review.

Any discrepancies between the two reviewers will be resolved by consensus, and in case of disagreement a third reviewer will be involved. When multiple articles are published based on the same study, we will select the one with the most recent. A PRISMA study selection flow chart will be provided (Moher et al., 2009) and we will include a table of excluded studies with the reasons for exclusion (Table 1).

**Table 1:** The reasons for exclusion

Excluded study (author, year)	The reason of excluded

#### 3.3.3 Data extraction and management

Two review authors will independently code and extract data from included studies. A coding sheet will be piloted on several studies and revised as necessary (see Table 2. Data extraction). Disagreements will be resolved by consulting a third review author with extensive content and methods expertise. Disagreements resolved by a third reviewer will be reported. Data and information will be extracted on: available characteristics of participants, exposure characteristics (marital transitions) and control conditions, research design, sample size, risk of bias and potential confounding factors (e.g., age, gender, education, and et al.), and outcomes. Extracted data will be stored electronically. Analysis will be conducted using RevMan5.3 and Stata 15.0 software.

**Table 2:** Data extraction

Baseline characteristics	Title
	Author, year
	Journal
	Study design
	Population location (geographical region)
	Total sample size
	Age (mean, SD)
	Gender (male), *n* (%)
	Follow up‐duration
	The transition duration (the length of time in transition)
	Neighborhood
	Social support
	Housing information
Exposures and control	Divorced, widowed, married, remarriage and unmarried
	Ascertainment of exposure (e.g., questionnaire)
Outcome	Name of outcome
	Definition of outcome
	Event and sample size
	Subgroup analysis (focus on age, gender, education, study design, duration of marital transitions and et al.)
	Type of effect size (RR, OR or MD)
	Effect size and 95% CI
	Covariates in fully adjusted model
Quality assessment	ROBINS

#### 3.3.4 Assessment of risk of bias in included studies

Two reviewers will use the Risk of Bias In Non‐randomized Studies (ROBINS) risk of bias tool to assess all observational studies (e.g., cross‐sectional, cohorts and case‐controls), This tool has been chosen as unlike other risk of bias tools, the ROBINS tool takes into account internal validity and attempts to mimic an RCT. This allows for more rigorous evaluation of bias that can arise from potential confounding of the effect, recall bias, differential misclassification of outcome and/or intervention status, attrition bias, reporting bias and detection bias. The tool contains seven items which are confounding, selection of participants, classification of interventions, deviations from interventions, missing data, measurement of outcomes and selection of reported result, and there are five categories of quality which are low, moderate, serious, critical and no information (Igelström et al., 2021; Sterne et al., 2016). We will provide a risk of bias table with the judgment and the justification of each item, and a summary graph of bias for each study and for all of them.

#### 3.3.5 Measures of treatment effect

Dichotomous data will be analyzed using numbers of events of each study, RRs or ORs, with 95% CIs, continuous data using mean and standard deviation, and the number of participants in each group.

Binary outcomes were presented as ORs with 95% CIs, the overall effect was pooled using a random‐effects model. We will pool data according to the adjusted ORs or unadjusted ORs (calculated by events and sample size) respectively. If the study reports the RRs, we will convert it to ORs. As for study designs, pooling data for all study designs, then we will conduct subgroup analysis according to the different types of study design.

#### 3.3.6 Unit of analysis issues

We will pay caution to ensure that the same group of participants is not included twice in a single meta‐analysis. In addition, paired data will be analyzed appropriately.

#### 3.3.7 Criteria for determination of independent findings

##### Dealing with missing data

In case of missing data, we will contact the authors at least twice to obtain these data, if correspondence details are available.

Where possible, we will calculate missing values (e.g., RR, 95% CI, and *p* values) from the available data, using the Review Manager 5 software (Cochrane Training, 2021; Higgins et al., 2019). If insufficient data are available to calculate missing values, we will only analyze the available data and describe the results from the studies with missing data narratively.

In the final review, the issue of the missing data and their potential impact on the findings will be discussed in the discussion section.

#### 3.3.8 Assessment of heterogeneity

Heterogeneity will be assessed first, through visual inspection of the forest plot and checking for overlap of CIs and second through the *χ* ^2^ test and *I* ^2^ statistics. The *χ* ^2^ test will be performed and the *I* ^2^ statistic will be calculated to quantify inconsistency across studies. For the *χ* ^2^ test, a *p* value of 0.10 will be used as a threshold for statistical significance. An *I* ^2^ threshold of 60% will be adopted. However, following the guidance of the Cochrane Handbook for Systematic Reviews of Intervention (Deeks et al., 2019), care will be taken in interpreting the results, should studies be few in number or have small sample sizes.

#### 3.3.9 Assessment of reporting biases

If 10 or more studies are identified, publication bias will be assessed through visual inspection of funnel plots. If the funnel plot shows asymmetry, a formal statistical Egger test will be performed. If there is evidence of funnel plot asymmetry from a test, we will attempt to distinguish the different possible reasons for this (nonreporting biases, poor methodological quality leading to spuriously inflated effects in smaller studies, true heterogeneity, artefactual, chance) (Page et al., 2019).

#### 3.3.10 Data synthesis

If two or more studies are identified that have investigated the effect of the same exposures on the same outcome, and data are sufficiently available, these data will be pooled and random effects meta‐analyses will be performed due to the expected between‐study variation, using the Review Manager 5 software. The Mantel–Haenszel method will be used for dichotomous outcomes, respectively. Meta‐analysis results will be visually presented in forest plots.

If the meta‐analysis is not possible, we will use the method of narrative synthesis of quantitative data, the data (e.g., events, sample size and point estimates) will be presented with forest plot and grouped by different outcomes and comparisons. For each comparison and outcome, a description of the synthesis findings would be provided, making clear the direction of outcome of each study (Campbell et al., 2020).

#### 3.3.11 Subgroup analysis and investigation of heterogeneity

If substantial statistical heterogeneity is detected, heterogeneity may be explored by conducting subgroup analyses or (if at least 10 studies are included in the meta‐analysis) by conducting meta‐regression to guard against potential issues of confounding (Deeks et al., 2019). Heterogeneity may occur due to:

1. Study design: We will include cohort, case‐control and cross‐sectional study in our systematic review, the results in different study design may be distinguishing.
2. Age: Different age group have different life experience, so as to different perception of marital changes (Rendall et al., 2011).
3. Gender: Men and women tend to seek their emotional support and reassurance may be various, and the knowledge about the process by which men and women adjust to marital change remains fragmentary, so when they experience marital change, the impact of gender on the association between marital transitions on health would be different (Strohschein et al., 2005; Walker & Luszcz, 2009).
4. Education: Because the ability to weather later‐life marital transitions may depend on the long arm of education acquired earlier in the life course, so this study plan to explore the subgroup of education level (Recksiedler & Stawski, 2019).
5. Geographical region: The impacts of marriage on people's life vary diametrically among regions where socio‐cultural backgrounds are dissimilar to each other (Fu & Noguchi, 2016).
6. Housing, neighborhood and social support: We hypothesis that stable housing, good neighbor relationships and extensive social support would weaken the impact of marital transitions on health in late life.
7. The duration of marital transitions: the long‐term impacts of marital transition on health are likely to differ substantially from short‐term impacts.

#### 3.3.12 Sensitivity analysis

Sensitivity analyses may be performed with respect to the quality of studies to test the robustness of the meta‐analysis by assessing whether results are not influenced by the inclusion or exclusion of low‐quality studies.

#### 3.3.13 Treatment of qualitative research

We do not plan to include qualitative research.

#### 3.3.14 Summary of findings and assessment of the certainty of the evidence

**Figure 1:**
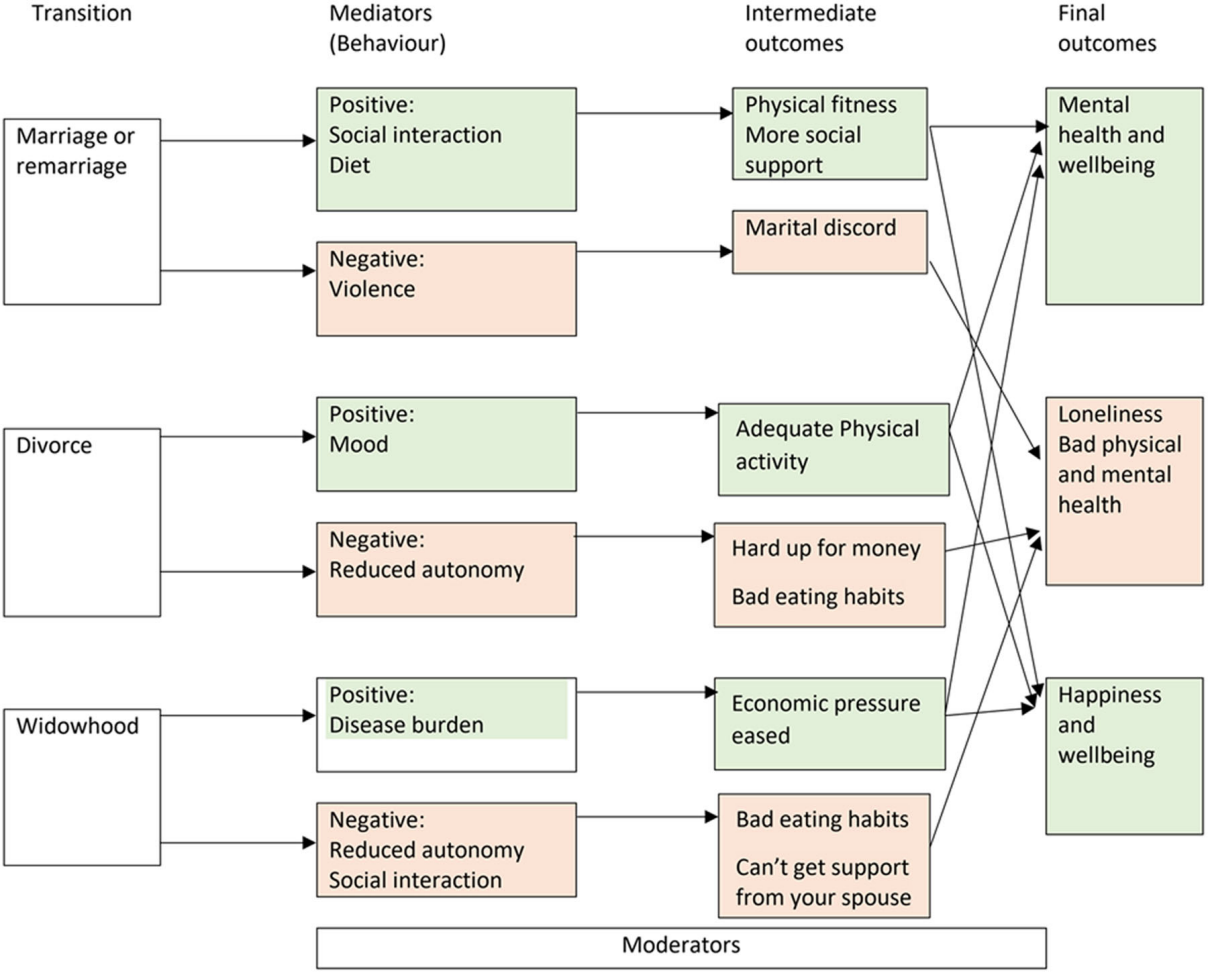
The conceptual framework of the study.

The GRADE (Grades of Recommendation, Assessment, Development, and Evaluation) system will be used to evaluate the certainty of the underlying evidence (Guyatt et al., 2008). The system classifies certainty of evidence as high, moderate, low, or very low (Brozek et al., 2009; Yang et al., 2013). Five of the eight criteria proposed in the GRADE method have the potential to decrease one's confidence in the correctness of the effect estimates: risk of bias, inconsistency of results across studies, indirectness of evidence, imprecision, and publication bias. Three further criteria are proposed that have the potential to increase this confidence: a large magnitude of effect with no plausible confounders, a dose–response gradient, and a conclusion that all plausible residual confounding would further support inferences regarding treatment effect. GRADE proposes these three criteria should be considered particularly in observational studies. (Guyatt et al., 2011) We will use the web page named Guideline Development Tool (https://gdt.gradepro.org/app/) to evaluate the certainty of evidence and calculate the absolute effects. A “Summary of findings” table will be provided in the review, this table will present the overall quality of the body of evidence according to GRADE criteria for the physical and mental health outcomes. In the summary of finding table, we will present the data of total sample but not subgroup for physical and mental health, and all the comparisons will be presented (Figure 1).

## CONTRIBUTIONS OF AUTHORS

J. L. drafted the protocol. All authors reviewed the draft and approved the final version.

- Content: Jing Li, Xu Hui, Kehu Yang
- Systematic review methods: Liang Yao, Peijing Yan
- Statistical analysis: Peijing Yan, Liang Yao
- Information retrieval: Jing Li, Xu Hui, Zhenxing Lu, Xiaocao Ren, Wenlong Yan

## DECLARATIONS OF INTEREST

The authors declare no conflict of interests.

## SOURCES OF SUPPORT

**Internal sources**

None.

**External sources**

This study is supported by the Major Project of the National Social Science Fund of China: Research on the Theoretical System, International Experience, and Chinese Path of Evidence‐based Social Science (NO.: 19ZDA14).

## Supporting information

Supporting information.
