# Supplementary material for: PROTOCOL: The association between marital transitions and physical and mental health in late life: A systematic review
Source: Campbell Syst Rev. 2022 Jun 8;18(2):e1252. doi: 10.1002/cl2.1252 (PMC9175064; doi:10.1002/cl2.1252)
Supplement: Supplementary file 1 — Supporting information. [file CL2-18-e1252-s001.docx]

Appendices

1 Pubmed ALL search strategy

From database inception to June 29, 2020

| **#** | **Searches** | **Results** |
| --- | --- | --- |
| #1 | "Aged"[MeSH Terms] OR "Retirement"[MeSH Terms] OR "Geriatrics"[MeSH Terms] OR "Frail Elderly"[MeSH Terms] | 3,396,842 |
| #2 | "aged"[Title/Abstract] OR "elderly"[Title/Abstract] OR "retirement"[Title/Abstract] OR "retired"[Title/Abstract] OR "pensioner"[Title/Abstract] OR "pensioning"[Title/Abstract] OR "pensioned"[Title/Abstract] OR "old people"[Title/Abstract] OR "older adult*"[Title/Abstract] OR "later life"[Title/Abstract] OR "end of life"[Title/Abstract] | 989,415 |
| #3 | #1 OR #2 | 3,938,991 |
| #4 | "Marriage"[MeSH Terms] OR "Spouses"[MeSH Terms] OR "Widowhood"[MeSH Terms] OR "Divorce"[MeSH Terms] OR "Single Person"[MeSH Terms] | 39,902 |
| #5 | "marital transition"[Title/Abstract] OR "marital change"[Title/Abstract] OR "marital dissolution"[Title/Abstract] OR "marriage crisis"[Title/Abstract] OR "marri*"[Title/Abstract] OR "divorc*"[Title/Abstract] OR "widow*"[Title/Abstract] OR "unmarried"[Title/Abstract] OR "become widowed"[Title/Abstract] OR "become divorced"[Title/Abstract] OR "become married"[Title/Abstract] OR "become remarried"[Title/Abstract] OR "transition to marri* "[Title/Abstract] OR "divorce transition"[Title/Abstract] OR "transition to widowhood "[Title/Abstract] OR "Newly widowed"[Title/Abstract] OR "recently widowed"[Title/Abstract] OR "newlywed"[Title/Abstract] OR "recently divorced"[Title/Abstract] OR "Newly divorced"[Title/Abstract] | 66,472 |
| #6 | #4 OR #5 | 89,424 |
| #7 | "Cohort Studies"[MeSH Terms] OR "Prospective Studies"[MeSH Terms] OR "Longitudinal Studies"[MeSH Terms] OR "Epidemiologic Studies"[MeSH Terms] OR "Cross-Sectional Studies"[MeSH Terms] OR "Observational Study"[Publication Type] OR "Case-Control Studies"[MeSH Terms] OR "Retrospective Studies"[MeSH Terms] | 2,913,930 |
| #8 | "cohort stud*"[Title/Abstract] OR "cohort*"[Title/Abstract] OR "cross sectional stud*"[Title/Abstract] OR "prevalence stud*"[Title/Abstract] OR "observational stud*"[Title/Abstract] OR "survey"[Title/Abstract] OR "case control stud*"[Title/Abstract] OR " Concurrent Stud*"[Title/Abstract] OR " Incidence Stud*"[Title/Abstract] OR "Disease Frequency Survey"[Title/Abstract] OR "Case-Comparison Stud*"[Title/Abstract] OR "Case Comparison Stud*"[Title/Abstract] OR "Case-Referent Stud*"[Title/Abstract] | 1,698,954 |
| #9 | #7 OR #8 | 3,768,743 |
| #10 | #3 AND #6 AND #9 | 12,192 |
